# High-Temperature SAW Wireless Strain Sensor with Langasite

**DOI:** 10.3390/s151128531

**Published:** 2015-11-11

**Authors:** Lin Shu, Bin Peng, Zhengbing Yang, Rui Wang, Senyang Deng, Xingzhao Liu

**Affiliations:** 1 State Key Laboratory of Electronic Thin Films and Integrated Devices, University of Electronic Science and Technology of China, Chengdu 610054, China; E-Mails: s89s89s@126.com (L.S.); jokerrui1992@163.com (R.W.); dengsenyang1990@163.com (S.D.); xzliu@uestc.edu.cn (X.L.); 2 China Gas Turbine Establishment, Jiangyou 621703, China; E-Mail: zbyang668@163.com

**Keywords:** langasite, SAW wireless sensor, high temperature, strain

## Abstract

Two Surface acoustic wave (SAW) resonators were fabricated on langasite substrates with Euler angle of (0°, 138.5°, 117°) and (0°, 138.5°, 27°). A dipole antenna was bonded to the prepared SAW resonator to form a wireless sensor. The characteristics of the SAW sensors were measured by wireless frequency domain interrogation methods from 20 °C to 600 °C. Different temperature behaviors of the sensors were observed. Strain sensing was achieved using a cantilever configuration. The sensors were measured under applied strain from 20 °C to 500 °C. The shift of the resonance frequency contributed merely by strain is extracted from the combined effects of temperature and strain. Both the strain factors of the two SAW sensors increase with rising ambient temperature, and the SAW sensor deposited on (0°, 138.5°, 117°) cut is more sensitive to applied strain. The measurement errors of the two sensors are also discussed. The relative errors of the two sensors are between 0.63% and 2.09%. Even at 500 °C, the hysteresis errors of the two sensors are less than 5%.

## 1. Introduction

Nowadays, strain measurements for structural health monitoring (SHM) are in great demand, particularly in the aerospace, automotive and energy industries [1]. Strain sensors in such systems should be able to provide information regarding the health of the structure and warn about any structure damage. Wireless sensing is advantageous when traditional wired strain gages are not convenient or sufficiently robust for rotating machine components or in harsh environments.

Dielectric and microwave cavity resonators that can operate at temperatures up to 700 °C have been used for wireless passive high temperature sensing [2,3]. Studies show the commercial prospects of these sensors, if microminiaturization can be achieved. Surface acoustic wave (SAW) sensors fabricated on high-temperature piezoelectric substrates have attracted considerable attention due to the properties of being passive and having high sensitivity, small size, low cost and good reproducibility [4].

SAW is very sensitive to the perturbations of the piezoelectric substrate. The variations of ambient temperature, deformation of surface structure or applied strain would result in a change in the resonance frequency of the SAW resonator. Based on the change in resonance frequency, the extent of temperature variation and structure deformation of the SAW sensor could be evaluated. There have been many SAW strain sensors developed for SHM [5,6,7]. However, due to the limitation of the substrate materials, the working temperature of these sensors is always limited to below 150 °C. Compared with conventional piezoelectric crystals, such as quartz, lithium niobate (LN), and zinc oxide (ZnO), whose operating temperature is limited to 300 °C, lanthanum gallium silicate (langasite, LGS) is a promising high-temperature piezoelectric materials [8] because of its excellent temperature stability, high piezoelectric coupling coefficient, high Q-factor and low acoustic loss. Furthermore, LGS has a SAW velocity of 2742 m/s, a high strain gauge factor, and a large electromechanical coupling coefficient of 0.32%, which is larger than that of Quartz (0.14%) [9]. Due to its excellent piezoelectric properties, LGS has attracted many attentions in developing advanced SAW high-temperature sensors [10]. Zhang reported the force-frequency effect of LGS SAW resonators [11]. Zheng [12] and Thiele [13] fabricated the high-temperature LGS SAW gas sensor. Another LGS SAW gas sensor for hash environment has been reported by David *et al.* [14]. However, to the best of our knowledge, there are no reports on a SAW high temperature strain sensor that can work at about 500 °C.

In this work, two SAW sensors based on different LGS cuts are measured with applied strain in wireless method. The maximum operating temperature reaches 500 °C and the maximum applied strain reaches about 300 με. The temperature and strain properties of the SAW sensors are investigated. The shift of the resonance frequency contributed merely by strain is extracted from the combined effects of temperature and strain. Furthermore, the measurement errors and hysteresis characteristics of the SAW sensors are also discussed.

## 2. Experimental Setup

In this work, the SAW sensors had a typical SAW resonator (SAWR) structure, which consisted of an interdigital transducer (IDT) and two reflector banks. Each IDT contained 101 equal-interval-finger electrodes with finger widths of 3 μm, yielding an acoustic wavelength of 12 μm. Each reflector bank contained 400 short-circuited gratings. The aperture W was 1200 μm. The electrodes were patterned by lift-off photolithography techniques on LGS substrates, which consist of a 10-nm-thick Ti adhesion layer and a 100-nm-thick Au film. Two SAW resonators with the same structure were fabricated on two different LGS substrates. The first SAW resonator (Device A) was fabricated on the LGS cut with Euler angle of (0°, 138.5°, 117°) and the second SAW resonator (Device B) was fabricated on the LGS cut with Euler angle of (0°, 138.5°, 27°). Before measurement, the SAW resonators were annealed at 600 °C for 30 min in pure N_2_ to improve its thermal stability.

**Figure 1:**
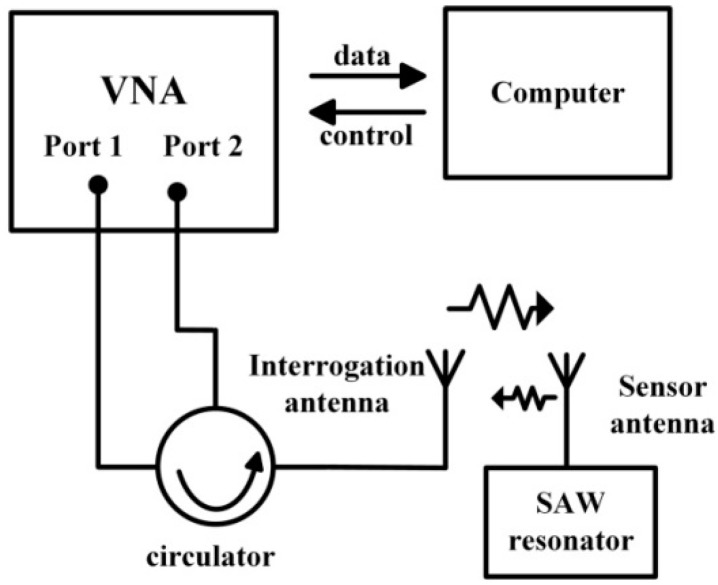
Diagram of the wireless measurement system.

In previous work on SAW sensors, various techniques have been used for wireless measurement. The frequency-domain measurement method was adopted in this study because it offers continuous detection and high resolution. For wireless measurements, the SAW devices were attached to a λ/4 dipole antenna that consists of two 30-cm-length conductor wires made of 0.5 mm diameter copper wire. The wireless interrogation distance reached 20 cm. A diagram of the measurement system is shown in Figure 1. The interrogating signal generated by a vector network analyzer (VNA, Agilent E5071b) was emitted through a circulator and an interrogation antenna. The reflective signal from the SAW sensor returned to the port 2 of the VNA through the interrogation antenna and the circulator. Finally, the measurement of the S_21_ parameter was recorded and processed by a computer in the format of group delay. With the measured group delay curve, the resonance frequency of the SAW sensor could be determined.

**Figure 2:**
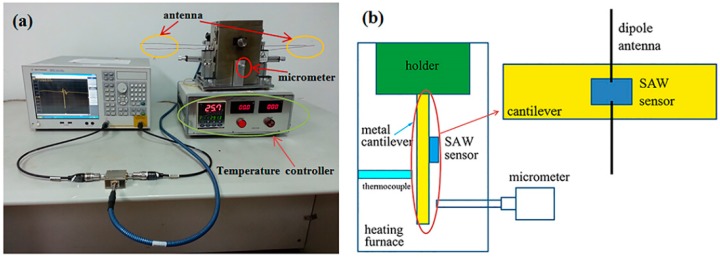
(**a**) Photo and (**b**) schematic diagram of the high temperature measurement setup.

In strain measurements, the SAW devices and dipole elements were fastened onto a metal cantilever with high-temperature ceramic adhesives (Huitian, C-2). The maximum operating temperature of the adhesives is 1730 °C, and the coefficient of thermal expansion (CTE) of the adhesives is about 8 × 10^−6^ /°C, which is similar to the CTE of ceramic. The cantilever configuration is shown in Figure 2. The dimension of the metal cantilever is 100 mm × 30 mm × 1 mm. The SAW devices are placed at the center of the cantilever. The cantilever is settled vertically with long axis normal to ground plane. One end of the cantilever is clamped by a holder and the other end is free. At the free end of the metal cantilever, a micrometer is used to force the cantilever bent so as to induce strain in the SAW devices. The induced strain can be calculated with the deflection of the cantilever [15], which is recorded at micrometer. The measuring precision of the micrometer is 0.01 mm. The induced strain can be written as:

(1) $$\varepsilon =\frac{3xdh}{2L^{3}}$$

where *d* is the deflection of the beam, *x* is the distance from the center of the device to the point where the load is applied, *h* is the thickness of the substrate, and *L* is the distance from the point where the substrate is clamped to the point where the load is applied. This experimental setup was placed inside a heating furnace except the micrometer, as shown in Figure 2. The inner size of the furnace cavity is about 100 mm × 70 mm × 140 mm. There is one small hole on each bilateral wall of the cavity. The copper wires of the antenna pass through the holes, so parts of the antenna are not contained in the cavity. Proper thermal insulation is taken to the cavity, especially around the holes. A thermocouple was located near the device to measure the device temperature accurately. With this experimental setup, the characteristics of the SAW sensor were measured at high temperatures, up to 600 °C.

## 3. Results and Discussions

**Figure 3:**
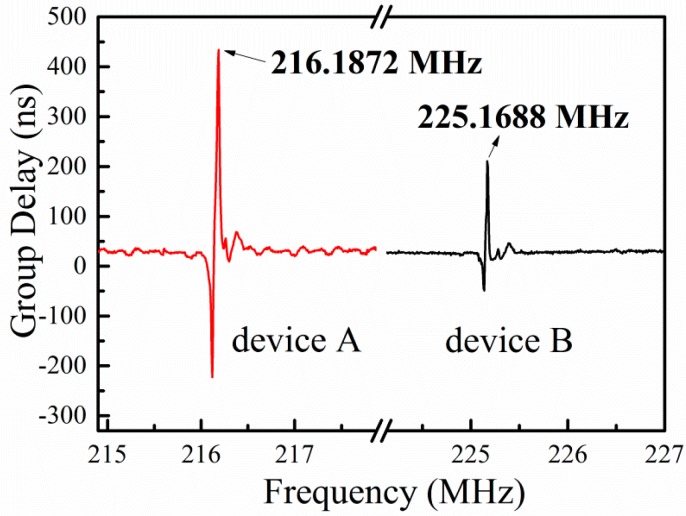
Group delay curves of the two devices at room temperature.

Figure 3 shows the measured group delay curves of the two SAW sensors at room temperature. In Figure 3, we can see that the group delay curve of each SAW sensor shows a sharp peak, which is originated from the resonance of the SAW resonator. It can be seen in Figure 3 that the resonance frequencies of Device A and Device B are 216.1872 MHz and 225.1688 MHz, respectively. The resonance frequency *f*_r_ of the SAW resonator can be expressed as:

(2) $$f_{r}=\nu /\lambda$$

where ν is the SAW velocity in the LGS substrate and λ is the wavelength of the IDT. In our devices, the wavelength is 12 μm. Then, with the Equation (2), the SAW velocity of Device A and Device B are 2594 m/s and 2702 m/s, respectively. It can be concluded that the SAW velocity is higher on (0°, 138.5°, 27°) cut than that on (0°, 138.5°, 117°) cut in LGS substrate. These results are very close to the reported data [9].

### 3.1. Temperature Response

**Figure 4:**
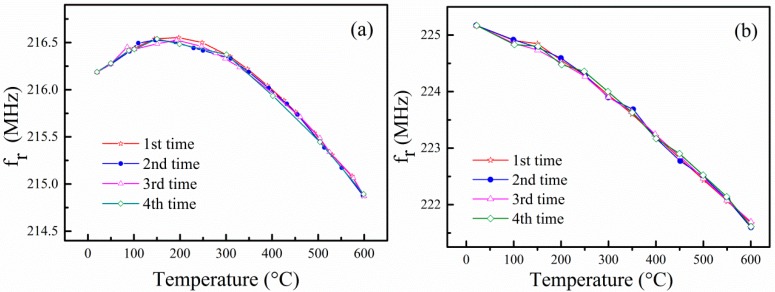
Resonance frequency of the devices as a function of temperature: (**a**) Device A and (**b**) Device B.

Figure 4 shows the resonance frequency of the devices as a function of temperature. The resonance frequency of the devices was measured from room temperature to 600 °C four times. It is observed that there is good reproducibility for the measured resonance frequency at each thermal cycling measurement, which indicates that the LGS SAW sensors are very stable below 600 °C. The stability of the SAW sensor is attributed to the stable Au-Ti electrodes and the annealing procedure before measurements. Au-Ti thin film electrodes are widely used for high temperature sensors for their excellent oxidation resistance and outstanding electrical properties [16]. The annealing process not only releases residual stresses but also eliminates voids in Au-Ti film electrodes, so as to improve the thermal stability of the prepared LGS SAW sensor.

**Figure 5:**
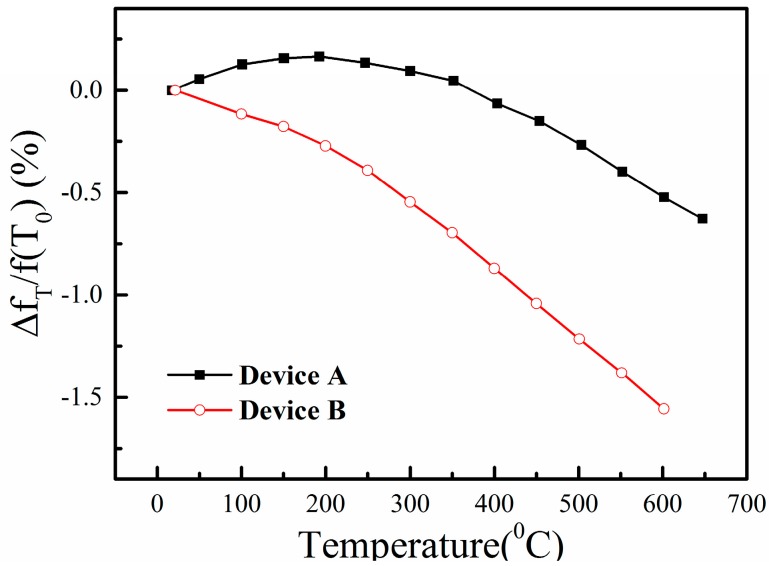
The relative changes of the resonance frequency as a function of temperature.

The measured relative changes of the SAW resonance frequency Δ*f*_T_/*f*_r_(*T*_0_) as a function of the temperature for the two SAW sensors are plotted in Figure 5. We can find that the relative resonance frequency change of Device B reaches −1.56% at 600 °C, while that of Device A is only −0.52%. The measured results of the two LGS sensors are in good agreement with Peng’s work on langasite temperature sensors [10], where the SAW velocity decrease at 600 °C was about 15 m/s (−0.5%) for LGS (0°, 138.5°, 117°) and 35 m/s (−1.2%) for LGS (0°, 138.5°, 27°). Furthermore, the relative resonance frequency change of Device A rises with the increasing of the temperature below 200 °C and then decreases with the further increasing of the temperature, while that of Device B decreases monotonically with the increasing of the temperature in the range of 20–600 °C. Furthermore, the Q factor of Device A decreases from 1292 to 403 from 20 °C to 600 °C, while the Q factor of Device B decreases from 1117 to 551 from 20 °C to 600 °C. These results suggest that SAW Device B is more suitable for temperature sensing because its dependence of the resonance frequency on the temperature is strong and monotonic.

The relative frequency variation with temperature of the SAW resonator *φ(T)* can be written as [17],

(3) $$\varphi (T)=\frac{\Delta f_{T}}{f_{r}(T_{0})}=\frac{f_{r}(T)-f_{r}(T_{0})}{f_{r}(T_{0})}$$

where *f_r_(T)* and the *f_r_(T_0_)* are the resonance frequencies of the devices at temperature *T* and reference temperature *T_0_*, respectively. 

In general, the thermal effects on the resonance frequency of the SAW resonator are due to the change in the length of the substrate and the substrate material parameter including density and elastic coefficients [17]. However, it should be noted that, in our experiments the thermal strain induced from the expansion of the cantileverand the thermal expansion mismatch between the sensor and cantilever also contribute to the shift of the resonance frequency because the SAW sensor is attached to the metal cantilever. Thus, we consider that the coefficient *φ(T)* includes all of the thermal influences of this sensing system at high temperature.

### 3.2. Strain Response

**Figure 6:**
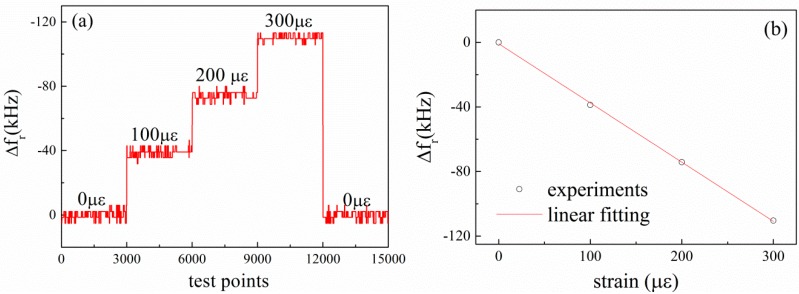
Resonance frequency shifts of Device A (**a**) under different strain loading sequences; and (**b**) as a function of the applied strain at room temperature.

To characterize the strain response of the prepared LGS SAW sensors, strain is applied to Device A in 100 με increments from 0 to 300 με, and returns to 0 με, while the temperature is held at room temperature. The applied strain as a function of the deflection was calibrated with resistive strain gauge before measurement. The measured resonance frequency shifts of Device A under different strain loading sequence are given in Figure 6a. In Figure 6a, we can see that the resonance frequency shift increases with the increasing of the applied strain. At each loading step, the resonance frequency changes very slightly. It is clear that the SAW response shows good correlation to the strain gauge. The mean resonance frequency shift of Device A as a function of the applied strain at room temperature is plotted in Figure 6b. It is observed that the resonance frequency shift decreases linearly with the applied strain. The resonance frequency shift decreases by 35 kHz when the applied strain increment is 100 με, which means that the resonance frequency shift is about 350 Hz per micro-strain for Device A. We can find this strain sensitivity coefficient is close to Wilson’s work [18]. Applied strain would not only change the acoustic wavelength, but also influences the elastic coefficients and density of the substrate materials [19]. Both of these parameters affect the acoustic wave propagation and are manifested in the changes in the velocity of the SAW devices, and then affect the resonance frequency [18].

Next, we discuss the temperature dependency of the SAW strain sensors at high temperature. When we consider the combined effects of the temperature and the applied strain on the SAW resonator, its resonance frequency can be expressed as [20]

(4) $$f_{r}(T,d)=f_{r}(T_{0},d=0)\times [1+\varphi (T)+\phi (d)]$$

where *f_r_(T,d)* is the resonance frequency of the SAW resonator when the ambient temperature is *T* and the deflection of the cantilever is d. *T*_0_ is the reference temperature and *ϕ(d)* is the strain factor of the SAW resonance frequency. Here, *ϕ(d)* only represents the frequency shift originated from the applied strain caused by the deflection *d*, because the frequency shift induced by the thermal effects of the LGS substrate and the metal cantilever is already included in *φ(T)*.

**Figure 7:**
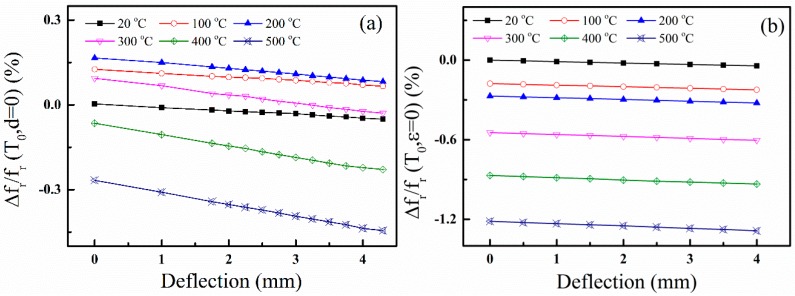
Dependence of the resonance frequency shift on the deflection at different temperatures: (**a**) Device A and (**b**) Device B.

The dependence of the resonance frequency shift on the deflection *d* at different temperature is shown in Figure 7. Here the resonance frequency shift is defined as:

(5) $$\frac{\Delta f_{r}}{f_{r}(T_{0},d=0)}=\frac{f_{r}(T,d)-f_{r}(T_{0},d=0)}{f_{r}(T_{0},d=0)}$$

In Figure 7, we can see that the resonance frequency shift decreases linearly with the increasing of the deflection of the cantilever, *i.e.*, the resonance frequency shift decreases with applied strain. At the same time, we can find that the resonance frequency shifts are due to the combined effects from temperature and strain. To extract the frequency change caused by the applied strain in Figure 7, the strain factor *ϕ(d)* is obtained from Equation (4) and expressed as

(6) $$\phi (d)=\frac{f_{r}(T,d)-f_{r}(T_{0},d=0)}{f_{r}(T_{0},d=0)}-\varphi (T)$$

**Figure 8:**
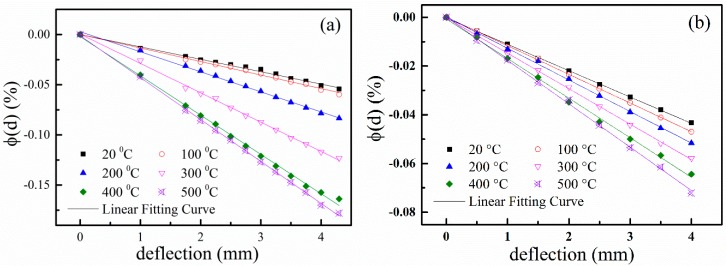
Dependence of *ϕ(d)* on the applied deflection at different temperature for (**a**) Device A and (**b**) Device B.

With the measured data, as shown in Figure 7 and Equation (6), the frequency shifts caused by strain are calculated and plotted in Figure 8. We can observe that *ϕ*(d) is linear to the deflection. Then, *ϕ(d)* can be expressed as

**Figure 9:**
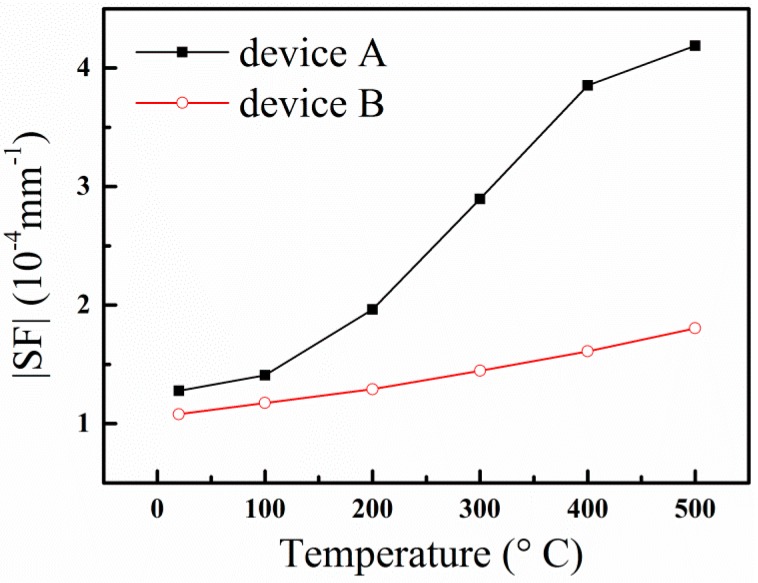
|SF| of Device A and Device B dependent on temperature.

(7) ϕ(d)=SF×d

where SF is the frequency sensitive factor of the SAW strain sensor, which is dependent on the applied deflection. From Equation (7), the slope of the frequency shift curves, as shown in Figure 8, is just the sensitive factor of the SAW strain sensor. We can find that the SF is different at different temperatures. The SFs of the two SAW sensors at different temperature are plotted in Figure 9.

In Figure 9, we can see that from room temperature to 100 °C, the sensitive factor SF of Device A is almost the same as that of Device B. However, it is obvious that Device A is more sensitive to the applied strain than Device B above 100 °C. At 500 °C, the absolute values of the SF of Device A and Device B are 4.19 × 10^−4^ mm^−1^ and 1.80 × 10^−4^ mm^−1^, respectively. These results show that the SAW is very sensitive to the strain on the LGS (0°, 138.5°, 117°) crystal cut. We can find that the SF of Device A and Device B are dependent on temperature. The |SF| values of Device A and Device B increase with increasing temperature, which indicates that the SAW sensors are more sensitive to strain at high temperatures. This is because the change of the elastic coefficients of the LGS substrate induced by applied strain at high temperatures is greater than that at room temperature [21]. At the same time, we can find that the SF values of Device A increase nonlinearly with increasing temperature, while the SF of Device B is linear with temperature. Though Device A has larger sensitivity to strain, it is difficult to compensate for temperature due to its nonlinear SF temperature dependency. However, it is easier to implement temperature compensation for Device B with its linear SF temperature dependency. The research of temperature compensation is very important for the application of SAW strain sensor. Further research on temperature compensation using compensating film techniques [22] is in process.

### 3.3. Error Analysis

The relative errors (*e*_L_) between the measurement data and the linear fitting line in strain measurements is defined as [7]

(8) $$e_{L}=\frac{\left|\Delta \phi (d)\right|}{Y_{FS}}\times 100\%$$

where *△ϕ(d)* is the maximum deviation between the measurement data and the fitting curve, and *Y_FS_* is the full Y-scale range of the measurement, which equals to *ϕ*(*T,d* = 4 mm)−*ϕ*(*T, d* = 0). The calculated *e*_L_ values of the two devices are presented in Table 1. The relative errors of the two sensors are between 0.63% and 2.09%, which indicates the linearity of the sensor is good in strain measurements. 

**Table 1:** The *e*_L_ of Device A and Device B at different temperatures.

T (°C)	20	100	200	300	400	500
*e*_L__A (%)	1.68	1.42	0.86	2.09	1.89	1.92
*e*_L__B (%)	1.08	1.11	1.23	0.63	1.59	1.66

The hysteresis error, which is the maximum deviation in output at any measurement value within the sensor’s specified range when approaching the point first with increasing and then with decreasing strain, is defined as [8]

(9) $$\delta_{Hm}=\frac{\left|\Delta H_{m}\right|}{Y_{FS}}\times 100\%$$

where ΔH_m_ is the maximum difference of the measured value in loading and unloading directions.

**Figure 10:**
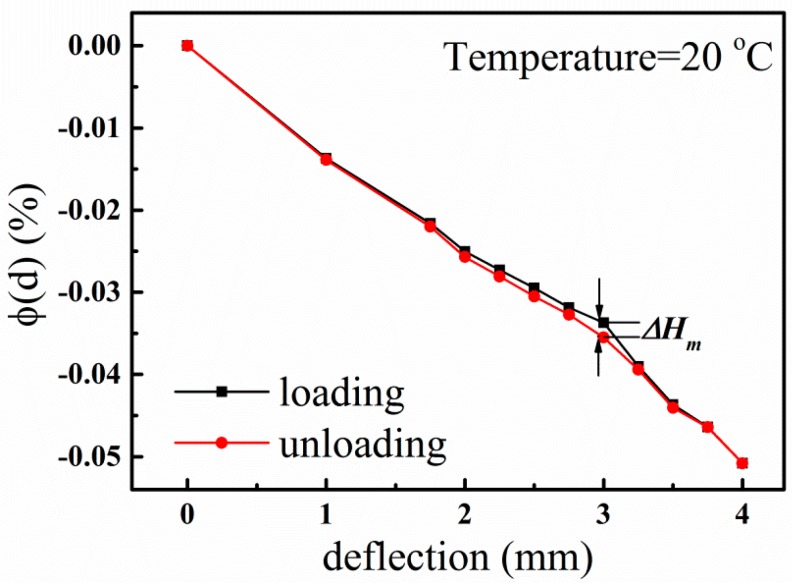
The hysteresis between loading and unloading direction of Device A at 20 °C.

In our measurements, hysteresis errors were observed (Figure 10). The *δ*_Hm_ of Device A and Device B are calculated and shown in Table 2. From Table 2, we can find that the hysteresis errors of the two devices increase with the rising of temperature, which indicates that the measurement error is bigger at high temperature. This is related to the adhesive used to install the sensor and the temperature conditions used to cure the adhesive. These hysteresis errors caused by temperature could be reduced by implementing an equivalent sensing system with two SAWR elements manufactured on a single die [23]. However, even at 500 °C, the *δ*_Hm_ of the two devices are still less than 5%, which is comparable with the results in [7] and indicates that these two sensors have good performance in high-temperature environments.

**Table 2:** The hysteresis of Device A and Device B at different temperatures.

T (°C)	20	100	200	300	400	500
*δ*_Hm__A (%)	3.55	3.62	3.40	4.02	4.27	4.53
*δ*_Hm__B (%)	2.48	2.04	2.58	3.66	3.97	4.79

## 4. Conclusions

As a conclusion, wireless SAW sensors were deposited on LGS substrate with Euler angle (0°, 138.5°, 27°) and (0°, 138.5°, 117°) cuts. From room temperature to 600 °C, the resonance frequency of the two SAW sensors under different applied strain was measured by wireless interrogation methods. The results show that the resonance frequency of the SAW sensor is dependent on both temperature and strain. The frequency shift caused by strain is extracted from the measurement data. The resonance frequency of the SAW sensor deposited on (0°, 138.5°, 27°) LGS crystal cuts decreases monotonically with the increasing of temperature in the range of 20–600 °C, while the resonance frequency of the other SAW sensor deposited on (0°, 138.5°, 117°) LGS crystal cuts varies with temperature non-monotonically, which indicates that the former SAW sensor is suitable for temperature sensing. The SAW sensor deposited on LGS substrate with Euler angle (0°, 138.5°, 117°) is more sensitive to applied strain. It should be noted that the change of the resonance frequency with strain is dependent on temperature, which makes it difficult to sense strain at high temperatures with one single SAW sensor. In future work, we propose a pair of SAW sensors that will be combined to make a multi-sensing system for sensing both temperature and strain. 
